# Utilization of cervical cancer screening services and its associated factors among primary school teachers in Ilala Municipality, Dar es Salaam, Tanzania

**DOI:** 10.1186/s12913-015-1206-4

**Published:** 2015-12-15

**Authors:** Neema Minja Kileo, Denna Michael, Nyasule Majura Neke, Candida Moshiro

**Affiliations:** World Health Organization, Tanzania Country Office, P O Box 9292 Dar es Salaam, Tanzania; National Institute for Medical Research, Mwanza Medical Research Center, P O Box 1452 Mwanza, Tanzania; Muhimbili University of Health and Allied Sciences (MUHAS), P O Box 67005 Dar Es Salaam, Tanzania

**Keywords:** Human Papiloma Virus, Screening, Cervical cancer and Primary school teachers

## Abstract

**Background:**

Worldwide cervical cancer is one of the more common forms of carcinoma among women, causing high morbidity and high mortality. Despite being a major health problem in Tanzania, screening services for cervical cancer are very limited, and uptake of those services is low. We therefore conducted a study to investigate utilization of cancer screening services, and its associated factors among female primary school teachers in Ilala Municipality, Dar es Salaam.

**Method:**

We conducted a cross-sectional study between May – August 2011 which involved 110 primary schools in Ilala Municipality in Dar es Salaam. Five hundred and twelve female primary school teachers were sampled using a two-stage cluster sampling procedure. Data on utilization of cervical cancer and risk factors were collected using a self-administered questionnaire. Proportional utilization of cervical cancer screening services was identified through a self report. Risk factors for services utilization were assessed using logistic regression analyses.

**Results:**

Out of 512 female primary school teachers, only 108 (21 %) reported to ever been screened for cervical cancer.

Utilization of cervical cancer screening services was 28 % among those aged 20–29, 22 % among married and 24 % among those with higher level of education. Women were more likely to utilize the cancer-screening service if they were multiparous (age-adjusted OR = 3.05, 95 % CI 1.15–8.06, *P* value 0.025), or reported more than one lifetime sexual partner (age-adjusted OR 2.17, 95 % CI 1.04–4.54, *P* value 0.038), or did not involve their spouse in making health decisions (adjusted OR 3.56, 95 % CI 2.05–6.18, *P* value <0.001).

**Conclusion:**

The study has demonstrated low level of utilization of cervical cancer screening service among female primary school teachers in Ilala munipality. Female primary school teachers with more than one previous pregnancy and those with more than one life-time sex partners were more likely to report utilization of the service. Spouse or partners support was an important factor in the utilization of cervical cancer screening service amongst the study population.

## Background

Cervical cancer, a complication of Human Papilloma Virus (HPV) infection, is one of the more common forms of carcinoma among women worldwide, accounting for about 12 % of all cancers in women [1]. While relatively low incidence rates prevail in Europe, North America and Japan (generally about 10/100,000 women), rates in sub-Saharan Africa are many times higher, as are the rates in other poorer countries in Latin America and the Caribbean, Melanesia, South and Southeast Asia [2]. Countries in East and Southern Africa have the highest reported age-standardized incidence rates per 100,000 women: Tanzania (68.6), Lesotho (61.6), Zambia (53.7) and Guinea (50.9) [2]. In 2002, cervical cancer was the second biggest cause of cancer-related mortality among women worldwide [2]. In sub Saharan Africa, cervical cancer is an important cause of mortality and morbidity in terms of amount of years of life lost (YLL) and years lived with disability (YLD) among women with child bearing age and it makes the largest contribution to Disability Adjusted Life Years (DALY’s) due cancer in this region [3].

In Tanzania, cervical cancer is a major health problem. It is the most common histological diagnosed cancer [4]. Cervical cancer ranks the first most frequent cancer among women in Tanzania, and the most frequent cancer among women between 15 and 44 years of age [5]. Cervical cancer screening is acknowledged currently as the most effective approach for cervical cancer control. One of the most important reasons for the huge disease burden of cervical cancer in developing countries is the lack of early detection and treatment of pre-cancerous lesions before they progress [6]. In developed countries between 40 % and 90 % of women are screened for cervical cancer [7], but in developing countries less than 5 % of women undergo cervical cancer screening. In 2001, Tanzania had the lowest numbers of women screened for cervical cancer per month in East Africa [8]. Several quantitative and qualitative studies have been carried out to determine factors contributing to low uptake of cervical cancer screening services in Kenya, Lesotho, Uganda and Zimbabwe [8–12]. In Tanzania, it is crucial to increase our knowledge of screening participation, and understand the factors affecting women’s participation in cervical cancer screening programs. This understanding will help to design and implement programs tailored to women’s needs that encourage women to seek screening, and thereby reduce the overall disease burden.

However, few studies have been carried out in Tanzania to determine uptake of cancer screening, and to establish factors influencing the utilization of cervical cancer screening services among women of reproductive age. Utilization of cervical cancer screening services and its associated factors has not been studied among female primary school teachers, which is the largest group of women employed as public servants in Tanzania. The government of Tanzania is in the process of developing a National Program for Human Papilloma Virus (HPV) vaccination as well as introducing Sexual and Reproductive Health Education in the primary school curricula. Primary school teachers will be an important channel to impart this knowledge to the younger generation and to the community as a whole.

We conducted this study to investigate utilization of cancer screening services, and its associated factors among female primary school teachers in Ilala Municipality, Dar es Salaam.

## Methods

### Study setting and population

**Fig. 1:**
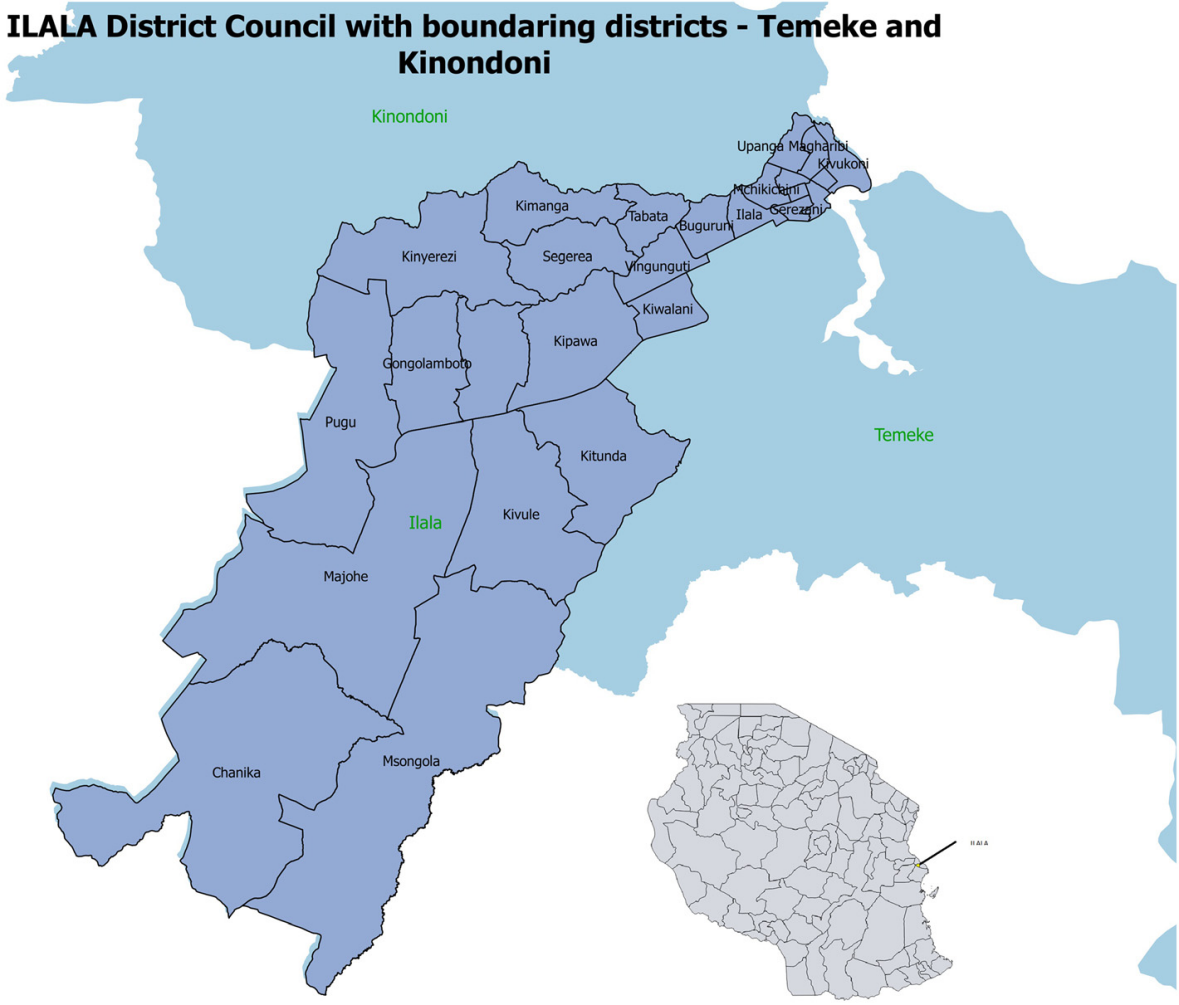
A map of Ilala District

This study was conducted in Ilala Municipality, which is one of 3 districts in Dar-es-Salaam, the commercial capital of Tanzania. In 2006, Dar-es-Salaam had a combined population of 2,822,140 [13], Ilala Municipality having a population of 634,924 people and 110 primary schools. See Fig. 1 for a map of Ilala district.

### Sampling and sample size

The target population for the study was female primary school teachers in Ilala Municipality. A two-stage cluster sampling procedure was used, with the first stage consisting of a list of all 110 primary schools in Ilala Municipality. Thirty primary schools were randomly selected from the list, using the lottery method. From each primary school, all available female teachers were selected to be included in the study. The minimum sample size was estimated to be 460 female primary school teachers, in order to estimate the proportion of women who have ever screened for cervical cancer with a precision of 4 %. However, sample size was calculated using the formula for single proportion: *N =* Z^2^P Q/ E^2^ [14] Where: *N* denotes sample size, Z denotes standard normal deviation, a constant set at the 95 % confidence interval, which is 1.96 and P denotes proportion of women who have ever screened for cervical cancer which is 15 % [15]. A margin of error (4 %) is denoted by letter E whilst and Q is calculated from 1-P. Ilala municipality was chosen because of its easy reach from coordinating office at Muhimbili Hospital and hence simplified other logistical costs including travel.

### Study design and data collection procedures

A two-stage cross-sectional study design was used, involving primary schools in Ilala Municipality. Individual level data was collected using self-administered questionnaires designed in English, translated into Kiswahili,, the medium of instruction in the participating primary schools. Swahili translation from English version was done in two steps. Firstly, English questionnaire was translated into Swahili. Then, another translator translated Swahili questionnaire back to English language. Investigators compared the two English versions of the questionnaires and approve the Swahili translated version if there was no distorted meaning between the two English versions. This process is called back-translation. The study questionnaire consisted of socio-demographic characteristics of respondents, knowledge about cervical cancer, attitudes towards cervical cancer screening, awareness on screening services available, screening practices and factors influencing utilization of cervical cancer screening services among female primary school teachers. Information on utilization of cervical cancer screening service was self-reported.

### Data and observations

Self reported use of cervical cancer screening services was the main outcome variable. The independent variables included: age group, level of education, marital status, number of previous pregnancies, and number of life-time sex partners and use of female contraceptives. Attitude and perception of cervical screening were also captured in the data and analyzed. However, most of independent variables chosen for this particular study were pre determined by previous studies [8–12].

### Quality control

Research assistants were trained on the subject matter of the study, the research tools and instruments. They were also trained on research ethics and the standard operating procedures and logistics for the study. They participated in pre-testing the questionnaire prior to the actual fieldwork, which was done few days before the execution of the study. The pre-testing of the questionnaire involved 25 females of reproductive age who were not primary school teachers, with the main aim to ensure that the questions were in a logical order, easily comprehended, and whether the field logistics were understood by the research assistants.

### Data processing and statistical analysis

EPI Data (Version 3.1) was used to enter the collected data into the computer, with double data entry to minimize data entry errors. Data cleaning was carried out to check for “forgotten” entries, consistency and outliers, and the final data exported to Stata for analysis using STATA version 12. Discrete and categorical data were summarized accordingly. Bivariate analyses were done to examine the relationship between independent variables and utilization of cervical cancer screening services. Statistical significance was assessed using Chi-square test. A p-value of less than 0.05 was considered statistically significant. A logistic regression analyses were performed to determine the independent variables that are significantly associated with utilization of cervical cancer screening services using odds ratios (OR) and 95 % confidence intervals (95 % CI). Potential confounders that changed the Odds ratios by more than 10 % were included in the final multivariate logistic regression model.

### Ethics statement

Ethical clearance was obtained from Ethics Review Board of the Muhimbili University of Health and Allied Sciences (MUHAS). Research permit was also sought and obtained from the Ilala Municipal authorities. Head Teachers from the 30 selected schools approved the study. Each participant provided written-Informed consent before commencement of any study related procedure. No identifying information was used in this study and all other collected information was kept strictly confidential.

## Results

A total of 512 female primary school teachers from 110 primary schools in Ilala were recruited into the study. The mean age was 38.2 ± 9.3 years with majority of them (42 %) in the age group 30 – 39. Most of the participants were married (80.9 %), and 84.3 % of them had primary school level of education. Primary school teachers who were multiparous accounted for nearly half of all study participants (Table 1).

**Table 1:** Baseline characteristics and utilization of cervical cancer screening services among female primary school teachers in Ilala Municipality, 2010

Variable	Total (*N =* 512)	Utilization of cervical cancer screening services (*n =* 108, 21 %)		
		*n*	%	*P* value
Age group (in years)				
20–29	106 (20.7 %)	30	28.3	
30–39	219 (42.8 %)	42	19.2	
40–49	102 (19.9 %)	17	16.7	
50 and above	85 (16.6 %)	19	22.4	0.166
Marital status				
Single	61 (12.0 %)	11	18	
Married	412 (80.9 %)	91	22.1	
Widowed/divorced	36 (7.0 %)	6	16.7	0.606
Education level				
Secondary	432 (84.3 %)	89	20.6	
Higher	80 (15.63 %)	19	23.8	0.526
Parity				
Zero parity	54 (10.5 %)	11	20.4	
Para one	139 (27.1)	23	16.6	
Multi-parityα	254 (49.6 %)	54	21.3	
Grand-multi parityβ	65 (12.7 %)	20	30.8	0.145
Life-time sex partners				
One	71 (14.0 %)	11	15.5	
Two or more	435 (86.0 %)	97	22.3	0.194
Ever used contraceptive				
Yes	311 (60.7)	72	23.2	
No	201 (39.3)	34	17.9	0.156
Know cervical that cancer is preventable				
Yes	347 (67.8)	98	28.2	
No	18 (3.5)	3	16.7	
I don’t know	147 (28.7)	7	4.8	<0.005
Involve spouse in making decision				
Yes	329 (77.8)	57	17.3	
No	94 (22.2)	40	42.5	<0.005
Procedures for screening is disgraceful				
Yes	46 (10.5)	8	17.4	
No	392 (89.5)	90	23	0.391

*N* denotes Total number, *n* denotes number in category, α = 2-4 previous pregnancies, β = 5 and above previous pregnancies

Statistical significance based on Chi-square *P* value. Bolded results are statistically significant at *P* ≤ 0.05. OR denotes Odds Ratios and 95 % CI denote 95 % confidence intervals

Out of 512 female primary school teachers, only 108 (21 %) reported to ever used cervical cancer screening. Self-reported utilization of cervical cancer screening services was highest (28 %) among those aged 20–29 years, among those who were married (22 %) and those with higher level of education (24 %). However, there was no association between self reported utilization of cancer screening services with age (*p =* 0.166), marital status (*p =* 0.606) and level of education (*p =* 0.526). Multiparous primary school teachers were more frequent among the participants (49.6 %), but parity was not found to be associated with utilization of cervical cancer screening services (Table 1).

There were 435 (86 %) primary school teachers who reported to have more than one lifetime sexual partners while 311(60.7 %) reported to have ever used contraceptive methods. Most participants knew that cervical cancer is preventable (67.8 %) and this was strongly associated with utilization of cervical cancer screening service (*p* < 001). Three hundred and twenty nine (77.8 %) of the participants involved their spouse in making decision to utilize cervical cancer screening and this was also found to be strongly associated with utilization of the service (*p* < 0.001).

Although parity of a female primary school teacher was not found to be associated with self-reported utilization of cervical cancer screening service on crude analysis, after adjusting for age, multiparous teachers were 3 times more likely to utilize the service compared to those with zero parity (age-adjusted OR = 3.05, 95 % CI 1.15 – 8.06, *P* value 0.025) (Table 2). Participants with more than one lifetime sexual partners were found to be 2 times more likely to utilize cervical cancer screening services compared to those with one lifetime sexual partner (age-adjusted OR 2.17, 95 % CI 1.04–4.54, *P* value 0.038). Self reported utilization of cervical cancer screening services was associated with age. Teachers who were aged 40–49 years less likely to utilize the screening services compared to those aged 20–29 years (OR = 0.51, 95 % CI 0.26–0.99, *P* value 0.047).

**Table 2:** Risk factors for utilization of cervical cancer screening services: Crude and age-adjusted analysis using logistic regression, 2010

Variable	Crude		Age - adjusted	
	OR (95 % CI)	P value	OR (95 % CI)	P value
Age group (in years)				
20–29	Ref			
30–39	0.60(0.35–1.04)	0.064	-	-
40–49	**0.51(0.26–0.99)**	**0.047**	-	-
50 and above	0.71(0.37–1.42)	0.351	-	-
Marital status				
Single	Ref		Ref	
Married	1.29(0.64–2.58)	0.472	1.77(0.83–3.75)	0.139
Widowed/divorced	0.91(0.30–2.72)	0.865	1.29(0.40–4.23)	0.671
Education level				
Secondary	Ref		Ref	
Higher	1.20(0.68–2.11)	0.526	1.28(0.72–2.28)	0.397
Parity				
Zero parity	Ref		Ref	
Para one	0.76(0.34–1.72)	0.532	0.90(0.40–2.02)	0.792
Multi-parity	1.05(0.51–2.19)	0.884	1.55(0.71–3.38)	0.275
Grand-multi parity	1.74 (074–4.09)	0.2	**3.05(1.15–8.06)**	0.025
Life-time sex partners				
One	Ref		Ref	
Two or more	1.57(0.79–3.10)	0.195	**2.17(1.04–4.54)**	**0.038**
Ever used contraceptives				
Yes	Ref		Ref	
No	0.72(0.46–1.13)	0.156	0.69(0.44–1.08)	0.105
Knows cervical cancer is preventable				
Yes	Ref		Ref	
No	0.51(0.14–1.80)	0.285	0.48(0.14–1.72)	0.261
I don’t know	0.13(0.05–0.29)	<0.001	**0.13(0.06–0.29)**	**<0.001**
Involve spouse in making decision				
Yes	Ref		Ref	
No	3.53(2.11–5.91)	<0.001	**3.73(2.22–6.26)**	**<0.001**
Procedures for screening is disgraceful				
Yes	Ref		Ref	
No	1.41(0.64–3.15)	0.392	1.35(0.60–3.05)	0.463

Bolded results are statistically significant at *P* ≤ 0.05 (Wald P value). OR denotes Odds Ratios and 95%CI denote 95 % confidence intervals

After adjusting for age, education level and use of contraceptives were not associated with self-report utilization of cervical cancer screening service. Our data also did not find that marital status is not associated with cervical cancer service utilization. The negative experience towards the cervical cancer screening reported by some female primary school teachers was not associated with the uptake of the service.

Female primary school teachers who did not involve their spouse in making the decision to seek health care were nearly 4 times more likely to screen for cervical cancer compared to those who involved their spouse in decision-making (age-adjusted OR 3.73, 95 % CI 2.22–6.26, *P* value <0.001). This effect persisted even after adjusting for all variables in multivariate analysis (adjusted OR 3.56, 95 % CI 2.05–6.18, *P* value <0.001). See Table 3

**Table 3:** Risk factors for utilization of cervical cancer screening service among female primary school teachers in Ilala Municipality, June 2010: A multivariable analysis

Variable	Adjusted OR	95 % CI	*P* value
Age group (in years)			
20–29	Ref	Ref	-
30–39	1.10	0.56–2.13	0.787
40–49	0.94	0.42–2.13	0.884
50 and above	2.74	1.18–6.38	0.020
Knows cervical cancer is preventable			
Yes	Ref	Ref	-
No	0.42	0.09–2.02	0.277
I don’t know	**0.12**	**0.06–0.31**	**<0.001**
Involve spouse in making decision			
Yes	Ref	Ref	-
No	**3.56**	**2.05–6.18**	**<0.001**
Procedures for screening is disgraceful			
Yes	Ref	Ref	-
No	2.22	0.89–5.56	0.089

Bolded results are statistically significant at *P* ≤ 0.05 (Wald *P* value). OR denotes Odds Ratios and 95 % CI denote 95 % confidence intervals

.

## Discussion

### Main findings

This study has shown low utilization of cervical cancer screening services among female primary school teachers in Ilala Municipality. Female primary school teachers with higher number of previous pregnancies and those with more than one life-time sex partners were more likely to report utilization of the service. Support from the spouse also played a role in influencing utilization cervical cancer screening services, as female primary school teachers who reported not involving their spouses in making decisions on seeking health care were more likely to report utilization of cervical cancer screening service.

### Comparing our findings with results from other studies

It is common to find low utilization of cervical cancer screening services among women in sub Saharan African Countries. In countries, where resources for health are inadequate, the level of education is low, and poverty is prevailing, low utilization of cervical cancer screening services can easily be explained by these factors. This is similar to what is depicted from our study. A previous study in Tanzania found that 96 % of interviewed women had never gone for screening [9], with similar findings from another study conducted in Nigeria among female health workers [13]. Despite the large proportion of female health workers being aware of cervical cancer and Pap smear, utilization of the Pap test among health workers was found to be very low [16, 17].

Two hospital-based studies in Kenya revealed a relatively low level of self reported cervical screening [9, 18]. However our study may not be closely comparable to the findings from the two Kenyan studies because of the differences in the study participants and the expected level of knowledge between the two groups. Primary school teachers were aware of HPV, and should have higher education, health awareness levels and social status than other citizens.

The current study revealed multipara female teachers had higher utilization of cervical cancer screening services contrary to the assumption that many numbers of previous pregnancies a woman had, make it more likely for her to have received health education and particularly on sexual and reproductive health many times compared to those with lower number of previous pregnancies. It is therefore important to target young women with higher parity in screening programs for cervical cancer with the knowledge that they are also at high risk of cervical cancer as reported in some studies [7, 17].

The current study showed that marital status was not associated with utilization of cervical cancer screening services. This is in contrary to a study done in India where it was found that married, divorced and widowed women were more likely than single women to utilize for cervical cancer screening services [19]. The current study has shown that middle-aged, primary school teachers were less likely to screen compared to younger ones. These findings are very similar to a study by Nene and his colleagues in 2011 when exploring determinant of women to participate in cervical cancer screening [19]. It was found that increasing age was associated with a decreased use of screening services (younger women were more likely to attend for screening than older women).

Although socioeconomic status may be an important determinant of the ability to pay for preventive services, one study found that women with higher income levels were less likely to participate in the visual inspection- based screening [20]. As far as socioeconomic status is concerned women with higher education level were more likely to participate in screening [16]. It has been shown in other studies that women are less likely to be screened when they do not understand what is being asked of them. However, similarly to other studies the current study has also revealed that women with large number of life-time sex partners tend to utilize cervical cancer screening services compared to those with few lifetime sex partners [20]. That can be explained by the fact that women with many lifetime partners may perceive themselves to be more at risk than the other given the extended HIV/AIDS education in countries that are more affected with the pandemic.

The current study has identified the role of domestic gender relations as a predictor for utilization of cervical cancer screening services among primary school teachers in Ilala Municipality. Similarly, domestic gender power relations were one of the factors which influence uptake of cervical cancer screening service in a rural community in Uganda [11] and in Serbia [21]. However, another qualitative study in Mexico noted that women may fail to seek screening because their male sexual partners may be opposing to a male provider giving the examination [22]. Alliance for Cervical Cancer Prevention (ACCP) research projects in Western Kenya, noted that many women did not seek cervical screening services because their husbands provided little support or were actively opposing [18].

Lazcano-Ponce EC et al conducted a population-based study in Mexico including 3,197 women between the ages of 15 and 49 years to determine the main factors associated with increased utilization of a cervical cancer screening program (CCSP) in a population with a high mortality rate due to cervical cancer [23]. Findings showed that women who had used two or more family planning methods and those who knew why the Pap test was given had a better history of Pap screening. On the contrary, our study did not show evidence that use of contraceptive among female primary school teachers could be associated with utilization of cancer screening services.

### Limitations of the study

The current study recruited female primary school teachers only. Female primary school teachers in an urban municipality like Ilala may neither be a good representation of women in the country nor primary school teachers in other rural parts of the country. This group of female teachers may be more literate and more health conscious than other groups of women elsewhere who are unemployed, less educated or in unstable employments. For these reasons, the study was prone to potential selection bias. The results need to be interpreted with caution, as they cannot be generalized to the general population in Ilala Municipality or in the country.

Another type of error that was eminent in this study was a reporting bias. Although a questionnaire for this study was a self-administered one, there was a possibility that some information was being under or over reported due to several reasons. For example, a failure to comprehend and understand the questions may lead to false responses. There were also social undesirability concerns due to some sensitive questions such as report on lifetime sex partners. However, we collected main outcome for this study by means of self-report. This increased the chance for misclassification of an outcome and therefore, it’s likely to impact on estimated crude and adjusted odds ratios.

The design for this study was cross-sectional in nature. Like other cross-sectional studies, establishment of temporality in association between exposure and outcome might be difficulty and therefore results need to be interpreted with cautions. The observed association between outcome and exposures in this study may also be due to reverse causality. For example it may be possible that those female primary school teachers were screened for cervical cancer in the past even before they had the pregnancies or before they encounter many lifetime sex partners. Finally, we did not collect information in all possible confounders for the association between outcome and exposures. This gave us little chance to control for other possible confounders that could explain utilization of cervical cancer screening services.

### Strengths of the study

Information from this study is pivotal to Tanzania local needs in particular informing the current government initiatives to introduce HPV vaccine for girls of age 9–13. Therefore primary school teachers’ understanding and uptake of cervical cancer screening services are of paramount importance. Findings from the study will shed light on developing an effective cervical cancer control program country especially the one that will involve schoolgirls.

### Recommendations for further studies

There is need for in-depth understanding on gender-related norms and social supports that operate in Tanzanian culture and how they influence uptake of cervical cancer screening service among women of childbearing age. There is also a need to explore differences in the utilization of cervical cancer among women in the general population versus those in the stable employment (such as female school teachers, female nurses and police women), urban versus rural and women with high versus low social economic status. We envisage the need to study the population on basis of house-to house rather than relying on hospital-based samples. Finally, we recommend further research work to explore the role and importance of female primary school teachers in shaping the delivery of HPV vaccine program to school-aged girls.

## Conclusion

This study was conducted at the right time when the country is strategizing to improve cervical cancer prevention and treatment services. Understanding and exploring utilization of cervical cancer screening service and its risk factors among female primary school teachers in Tanzania is important to give some light to the broad efforts of preventing and treatment of cervical cancer. This will provide useful information to the government and other stakeholders whilst they formulate effective strategies and programs that will address factors influencing utilization of cervical cancer screening services so as to prevent the alarming morbidity and mortality rates of cervical cancer in Tanzania. In order to promote uptake of HPV vaccination program among school-girls, program managers need to ensure that primary school teachers have a proper understanding on the matter and that they have good experience of the benefits associated with screening for cervical cancer for an effective control for cervical cancer. That is because primary school teachers are the ambassadors of all of the interventions which involve young females who are in primary school hence they can be used as such to promote and improve uptake of these services. The study has demonstrated low level of utilization of cervical cancer screening service in this important group and some determinants that need to be taken into account when designing and implementing control program in primary schools.
